# Peri-implantitis: Etiology, prevention and management strategies

**DOI:** 10.6026/973206300212753

**Published:** 2025-08-31

**Authors:** Piyush Javiya, Seyed Mohammad Elyas Hashemi Zadeh, Neelam Chandwani, Mirella Vaz, Manish Kumar Pandit, Rashmi Laddha

**Affiliations:** 1 Department of Prosthodontics and Crown & Bridge, K. M. Shah Dental College & Hospital, Sumandeep Vidyapeeth Deemed to be University, Piparia, Waghodia, Vadodara, Gujarat, India; 2 Department of Cariology and Comprehensive Care, NYU College of Dentistry, New York, USA; 3 Department of Dentistry, All India Institute of Medical Sciences Nagpur, Maharashtra, India; 4 Department of Prosthodontics and Crown and Bridge, Bharati Vidyapeeth (Deemed to be University) Dental College and Hospital, Navi Mumbai, Maharashtra, India; 5 Department of Oral and Maxillofacial Surgery, Shri Balaji Institute of Dental Sciences, Raipur, Chhattisgarh, India; 6 Department of Periodontics, Dr. RR Kambe Dental College and Hospital, Akola, Maharashtra, India

**Keywords:** Peri-implantitis, dental implants, biofilm, risk factors, implant maintenance, regenerative therapy, mucositis, implant surface, antibiotics, laser therapy

## Abstract

Peri-implantitis represents a biofilm-associated pathologic condition characterized by inflammation and progressive bone loss around osseointegrated dental implants, which is a major risk to the survival of the implant. The etiology of peri-implantitis as a multifactorial process that includes microbial colonization, host susceptibility, biomechanical overload and systemic factors such as diabetes and smoking is analyzed in this systematic review. Preventive measures such as aggressive oral hygiene protocols, patient education and surface modification of implants are highlighted for their risk prevention potential. Management strategies involve both non-surgical interventions-debridement, local antibiotics, and laser phototherapy and surgery, resective and regenerative strategies to the severity of the disease. Comprehensive understanding of peri-implantitis pathogenesis and evidence-based treatment is essential in optimizing implant success and patient experience.

## Background:

Peri-implant disease is a severe complication after implant surgery or treatment, influencing the surrounding tissues. Distinct and continuous clinical checkups, as well as the removal of causative factors such as smoking and periodontitis, are effective precautionary measures against peri-implant complications. The primary aim of this review is to provide a summary of the current literature regarding the etiology and prevention of peri-implant disease for healthcare providers [1]. Peri-implant inflammations represent serious conditions after dental implant treatment, affecting both the surrounding hard and soft tissue [2]. Dental implants now form the backbone of current restorative dentistry, providing a long-lasting and functionally stable option for edentulous patients and those with failing dentition. Their popularity and success stem largely from their high survival rates, biocompatibility, and osseointegration with the host bone [3]. Nevertheless, the long-term success of dental implants is increasingly challenged by biologic complications, particularly peri-implant diseases. Of these, peri-implantitis is of great concern, identified as a severe, progressive inflammatory disease of the soft and hard tissues around a functional osseointegrated implant. It is clinically characterized by bleeding on probing, suppuration, increased probing depth, and radiographic crestal bone loss after the initial bone remodeling [4]. The prevalence of peri-implantitis has been reported as highly variable, ranging from 10% to more than 40%, depending on diagnostic criteria, patient population, and length of follow-up. Peri-implantitis is considered the implant counterpart of periodontitis, with numerous overlapping etiological and risk factors. It usually arises from peri-implant mucositis, a reversible inflammatory process limited to the peri-implant mucosa [5]. Without timely intervention, the disease progresses to peri-implantitis, resulting in irreversible bone damage and eventual implant loss. The etiopathogenesis of peri-implantitis is multifactorial and involves complex mechanisms. Biofilm accumulation by microorganisms remains the chief precipitating factor, provoking a host-mediated inflammatory-immune response [6]. Other risk factors include poor oral hygiene, a history of periodontitis, systemic diseases such as uncontrolled diabetes mellitus, genetic susceptibility, smoking, excessive cement, ill-fitting prosthetic design, and mechanical overloading. Certain implant surface characteristics and implant-abutment connections may also modulate susceptibility [7]. Because peri-implantitis is often asymptomatic in its early phases and can progress silently, prevention and early detection are critical. Preventive measures emphasize meticulous plaque control, professional maintenance, and patient education [8]. Implant surface modifications, such as nanoscale coatings and hydrophilic treatments, have also been suggested to reduce bacterial adherence and improve soft-tissue integration. From a management perspective, non-surgical treatments including mechanical debridement, adjunctive use of antiseptics or antibiotics, and laser therapy are commonly employed in early stages [9]. However, their effectiveness diminishes in moderate to advanced lesions with extensive bone loss, where surgical interventions such as resective or regenerative procedures involving bone grafts and membranes become necessary. Therefore, it is of interest to systematically review the etiology, prevention strategies, and treatment of peri-implantitis by combining findings from high-quality, peer-reviewed literature.

## Materials and Methods:

This systematic review was designed and conducted according to Preferred Reporting Items for Systematic Reviews and Meta-Analyses (PRISMA) 2020 guidelines to ensure methodological transparency and reproducibility. The main purpose was to identify, critically evaluate, and synthesize clinical evidence about the etiology, prevention, and treatment of peri-implantitis in human subjects. The process was not registered prospectively, but the process had rigorous inclusion, exclusion, and reporting criteria. A systematic electronic search of four major scientific databases including PubMed, Scopus, Embase, and the Cochrane Library was done. Search strategy comprised of Medical Subject Headings (MeSH) and free-text terms such as but not limited to: "peri-implantitis," "peri-implant disease," "dental implants," "implant failure," "risk factors," "biofilm," "mucositis," "implant surface," "implant debridement," "laser therapy," "antibiotics," "regenerative therapy," and "management."." Boolean operators (AND, OR) were applied for narrowing down the search. The search was restricted to studies from January 2010 to March 2025 that was published in the English language. The included articles' reference lists and relevant reviews were hand-searched for extra eligible studies not picked up by electronic databases to ensure completeness.

The inclusion criteria were clearly defined: (1) human clinical studies including randomized controlled trials (RCTs), controlled clinical trials, prospective and retrospective cohort studies, and case-control studies; (2) studies addressing the etiology, risk factors, prevention, or treatment of peri-implantitis; (3) studies with clearly defined diagnostic criteria and measurable clinical outcomes; and (4) studies with a minimum follow-up period of 6 months in case of interventional trials. Exclusion criteria included: (1) animal studies or in vitro investigations, (2) case reports, case series with fewer than 10 participants, narrative reviews, expert opinions, or conference abstracts without peer-reviewed data; (3) studies focusing solely on peri-implant mucositis without bone loss; and (4) non-English publications. Two independent reviewers (Author A and Author B) screened all identified titles and abstracts for eligibility using predefined inclusion and exclusion criteria. Discrepancies during the selection process were resolved by consensus or by consulting a third reviewer (Author C). Full-text articles of selected abstracts were retrieved and assessed in detail for final inclusion. A standardized data extraction sheet was developed to collect relevant information from each included study, including author name, year of publication, country, study design, sample size, characteristics of the patient population, diagnostic criteria for peri-implantitis, type of intervention or preventive measure, duration of follow-up, and primary outcomes assessed.

## Results:

Microbial colonization continually surfaced as the most substantial underlying cause of peri-implantitis and biofilms were populated with Gram-negative anaerobic bacteria such as Porphyromonas gingivalis, Treponema denticola, and Tannerella forsythia, which activated a destructive inflammatory response around the implant. Other contributing causes included mechanical stress with occlusal overload, micromovements from the implant-abutment junction, and potential foreign body reactions to particles left on the implant surface. Risk factors demonstrated a multifactorial patient-specific aspect. Historical periodontal disease, smoking, diabetic patients exhibiting poor glycemic control, poor oral hygiene and excess restorative cement, prosthetic designs impeding plaque removal, type of mucosa type with not enough keratinized mucosa all had significant correlations to a higher occurrence of peri-implantitis. Implant surface features, specifically rough, and subcrestally positioned implants also had an increased ability for microbial retention. Not having a regular professional maintenance program also led to further disease progression. Preventive strategies suggested the integration of early risk assessment, individualized oral hygiene programs, and regular maintenance therapy. Patients with long-term involvement in the supportive care program showed a significantly reduced incidence and severity of peri-implantitis. Intentional modifications to implant surfaces and individuals use of hydrophilic or anti-bacterial coatings are desirable methods for limiting early biofilm attachment, but these results primarily come from laboratory or short-term stories. Treatment outcomes varied by stage of disease. Non-surgical modalities - mechanical debridement, antiseptics, localized antibiotics, and laser therapy provided limited benefit in early disease but with extensive lesions and extensive bone loss, surgical procedures were typically required. Guided bone regeneration augmentation techniques resulted in better bone fill compared to resective treatments. Adjunctive methods like probiotics, photodynamic therapy, and host-modulation provided supportive but limited additional outcomes. The findings are summarized in Table 1 (see PDF), provides an overview of key studies addressing etiology, prevention, and management of peri-implantitis.

## Discussion:

Peri-implantitis has developed into one of the most serious biological complications in today's implantology practice. Not only does it threaten the long-term survival of an implant, but it also contributes to the retreatment load. The findings from these systematic review recent studies confirm its multifactorial and complex nature, as an approach to etiology, prevention, and therapy has to be clinical, multidimensional, and individualized [10]. Development of bacterial biofilm as the main etiologic factor is well known to have microbial communities as those found in chronic periodontitis. The peri-implant sulcus in affected individuals appears to be dominated by anaerobic Gram-negative pathogens like, but not limited to, Porphyromonas gingivalis and Treponema denticola, which are associated with a destructively escalated inflammatory response leading to progressive peri-implant bone loss [11]. Microbial colonization is a requirement, but does not account for initiation or severity. Susceptibility of the Host through, but not limited to, genes, systemic conditions like diabetes mellitus, and behavior like smoking are contributory [12]. Mechanical factors like occlusal overload, microgap leakage, and poorly constructed prostheses can increase tissue destruction of dento-periodontal origin in inflammation. Past periodontitis has always emerged as one of the strongest predictors of peri-implantitis which serves as further evidence that those likely to suffer periodontal tissue destruction remain in jeopardy even with the completion of full mouth rehabilitation [13]. Additionally, decreased levels of plaque control, whether by poor maintenance or faulty prosthetic design will also increase prevalence. Further organizational risk enhancers include excess cement and inadequate keratinized mucosa. Data indicates routine professional maintenance and early detection as some of the best methods to prevent disease diagnosis progression. Well-organized supported-care that includes managing bleeding on probing, maintaining probing depth, and periodic radiographs of crestal bone levels, were all strongly suggested [14]. Even in high-risk patients, the implant can remain disease-free as long as they enter an individualized prevention.

New forms of prevention - nanostructured implant surfaces, antimicrobial surfaces, and biomimetic modifications - have promise for preventing bacterial colonization, but most studies remain preliminary. Peri-implantitis treatment remains a highly controversial topic in implantology. In non-surgical cases with shallow to moderate pockets, treatment with mechanical debridement, antiseptic mouths rinses, and localized antibiotics can produce minimal gains; however, it is usually ineffective in stopping the disease in advanced peri-implantitis cases [15]. Adjunctive therapies such as lasers and air-abrasive systems have also been studied, but there is not always evidence that they have been more effective than traditional procedures. Surgical intervention is usually warranted in cases that present with severe bone loss. Often, regenerative procedures such as guided bone regeneration in well-contained defects have better outcomes than resective procedures because they have the potential for the restoration of lost bone and potentially better prognoses [16]. Newer therapies such as host modulation, photodynamic therapy, and probiotics are under investigation. These therapy options look to provide beneficial support; however, they should be used in conjunction with mechanical therapy, and require further study with larger, longer-term clinical trials before they are able to be added to the standard of care. A major limitation throughout the literature is the lack of standard diagnostic criteria, which makes it difficult to compare studies and develop guidelines. In addition, with variability in defining disease thresholds, inconsistent follow-up times, and inconsistent outcome measures, these inconsistencies are also problematic [17]. There is also a lack of long-term randomized controlled trials given clinical practice and the high prevalence of peri-implantitis. While this review did a thorough search and focused on human clinical studies with bias assessment, the findings are still qualitative due to the heterogeneity of studies between them and including only English and excluded grey literature.

## Conclusion:

Peri-implantitis is a multifactorial disease requiring timely diagnosis, tailored preventive strategies, and evidence-based management protocols to preserve implant function and longevity. While microbial biofilm remains the primary trigger, successful treatment demands a comprehensive approach that addresses both local and systemic risk factors. Continued research and clinical standardization are essential to optimize therapeutic outcomes and reduce the global burden of implant-related complications.
